# Combining entity co-occurrence with specialized word embeddings to measure entity relation in Alzheimer’s disease

**DOI:** 10.1186/s12911-019-0934-5

**Published:** 2019-12-05

**Authors:** Go Eun Heo, Qing Xie, Min Song, Jeong-Hoon Lee

**Affiliations:** 1 Department of Library and Information Science, Yonsei University, 50 Yonsei-ro Seodaemun-gu, Seoul, 03722 Republic of Korea; 2 Department of Creative IT Engineering, POSTECH, 77 Cheongam-ro Nam-gu, Pohang, Gyeongbuk 37673 Republic of Korea

**Keywords:** Information extraction, Semantic relatedness, Ranking algorithm, Knowledge discovery, Alzheimer’s disease

## Abstract

**Background:**

Extracting useful information from biomedical literature plays an important role in the development of modern medicine. In natural language processing, there have been rigorous attempts to find meaningful relationships between entities automatically by co-occurrence-based methods. It has been increasingly important to understand whether relationships exist, and if so how strong, between any two entities extracted from a large number of texts. One of the defining methods is to measure semantic similarity and relatedness between two entities.

**Methods:**

We propose a hybrid ranking method that combines a co-occurrence approach considering both direct and indirect entity pair relationship with specialized word embeddings for measuring the relatedness of two entities.

**Results:**

We evaluate the proposed ranking method comparatively with other well-known methods such as co-occurrence, Word2Vec, COALS (Correlated Occurrence Analog to Lexical Semantics), and random indexing by calculating top-ranked entities related to Alzheimer’s disease. In addition, we analyze gene, pathway, and gene–phenotype relationships. Overall, the proposed method tends to find more hidden relationships than the other methods.

**Conclusion:**

Our proposed method is able to select more useful related entities that not only highly co-occur but also have more indirect relations for the target entity. In pathway analysis, our proposed method shows superior performance at identifying (functional) cross clustering and higher-level pathways. Our proposed method, resulting from phenotype analysis, has an advantage in identifying the common genotype relating to phenotypes from biological literature.

## Background

With the recent exponential growth of biomedical literatures, extracting useful information from these literatures has come to play an important role in the development of modern medicine. In the biomedical domain, information extraction (IE) is focused mainly on automatically identifying entities and their relationships from biomedical literatures as an aspect of natural language processing (NLP). Traditionally, detecting biomedical relationships between entities commonly involves adopting co-occurrence methods, which are based on the assumption that if two entities appear in the same sentence, paragraph, or abstract, these entities would be relevant to each other and helpful for biomedical knowledge discovery such as gene–gene interaction and gene–drug association. However, co-occurrence methods have posed the problem of generating many false positive relations, since they do not consider contextual information in a specific text [1].

In addition to simple co-occurrence-based approaches to measuring the relationship between entities, rule-based methods using syntactic patterns [2–5] and machine learning methods [6, 7] have been proposed in order to tackle this false positive issue. Measures of semantic similarity and relatedness have been developed to identify ontological relationships between two entities, such as WordNet [8] and UMLS (Unified Medical Language System) [9]. Recently, models of semantic word representations, or word embeddings, have been developed constructing semantic spaces based on large-scale corpora. This line of research adopts deep learning approaches [10–16] such as Word2Vec [17] for automatically learning optimal feature representation. However, these studies focus only on learning word embeddings by maximizing raw-text probability, which does not perfectly capture both similarity and relatedness [18].

As indicated by previous studies [18–21], incorporating two or more knowledge sources (e.g. thesaurus, ontology, and corpus) into word embedding approaches can produce better results for ranking the results for relationships between two entities. The present paper was motivated by the concept of utilizing knowledge sources for enriching word embeddings. To our best knowledge, no attempt has previously been made to combine word embedding based on multiple knowledge resources with co-occurrence of entity pairs, while classifying the type of relation by reflecting contextual information in biomedical literature. Moreover, there is no previous study that considers both direct and indirect relationships of entity pairs when calculating co-occurrence of entity pairs.

Therefore, in this study, we propose a hybrid semantic relatedness algorithm for biological knowledge discovery. Our proposed method combines co-occurrence between entities with specialized word embeddings [18] to calculate the semantic similarity of two entities by capturing both similarity and relatedness for semantic words, learning from both a corpus and a thesaurus. In the proposed method, we also consider both direct and indirect scores for each entity pair so as to find a more complex relationship considering not only explicit but also hidden relationships. We select Alzheimer’s disease (AD) as a case study for analysis and evaluation. Alzheimer’s disease is a degenerative brain disorder, whose cause is hard to diagnose accurately. As the number of AD patients has increased, researchers have striven by means of medical experiments and literature analysis to understand the disease’s pathophysiology so as to improve its diagnosis and treatment. For entity extraction, we used two approaches, PKDE4J [22] and SemRep [23]. PKDE4J is an integrated system designed to extract entity and relation from unstructured biomedical text corpora, whereas SemRep, a UMLS-based entity and relation extraction application, can identify semantic relationships in biomedical literatures. To evaluate the performance of the proposed method, we compared it with several well-accepted techniques, namely co-occurrence, Word2Vec [17], COALS (Correlated Occurrence Analog to Lexical Semantics) [24], and random indexing (RI) [25]. In addition, to evaluate the usefulness of the proposed method for other types of knowledge discovery, we conducted the following analyses 1) pathways analysis on the Reactome Pathway database [26] and 2) gene–phenotype relationships analysis on OMIM (Online Mendelian Inheritance in Man) [27]. Overall, the proposed method is able to identify more related genes for pathways than the other methods by differentiating rankings for each gene. The proposed method also finds genes like APOE, which is strongly associated with familial early-onset AD and coronary heart disease [28], through analyses of AD-related genes and the gene–phenotype relationship.

## Methods

**Fig. 1:**
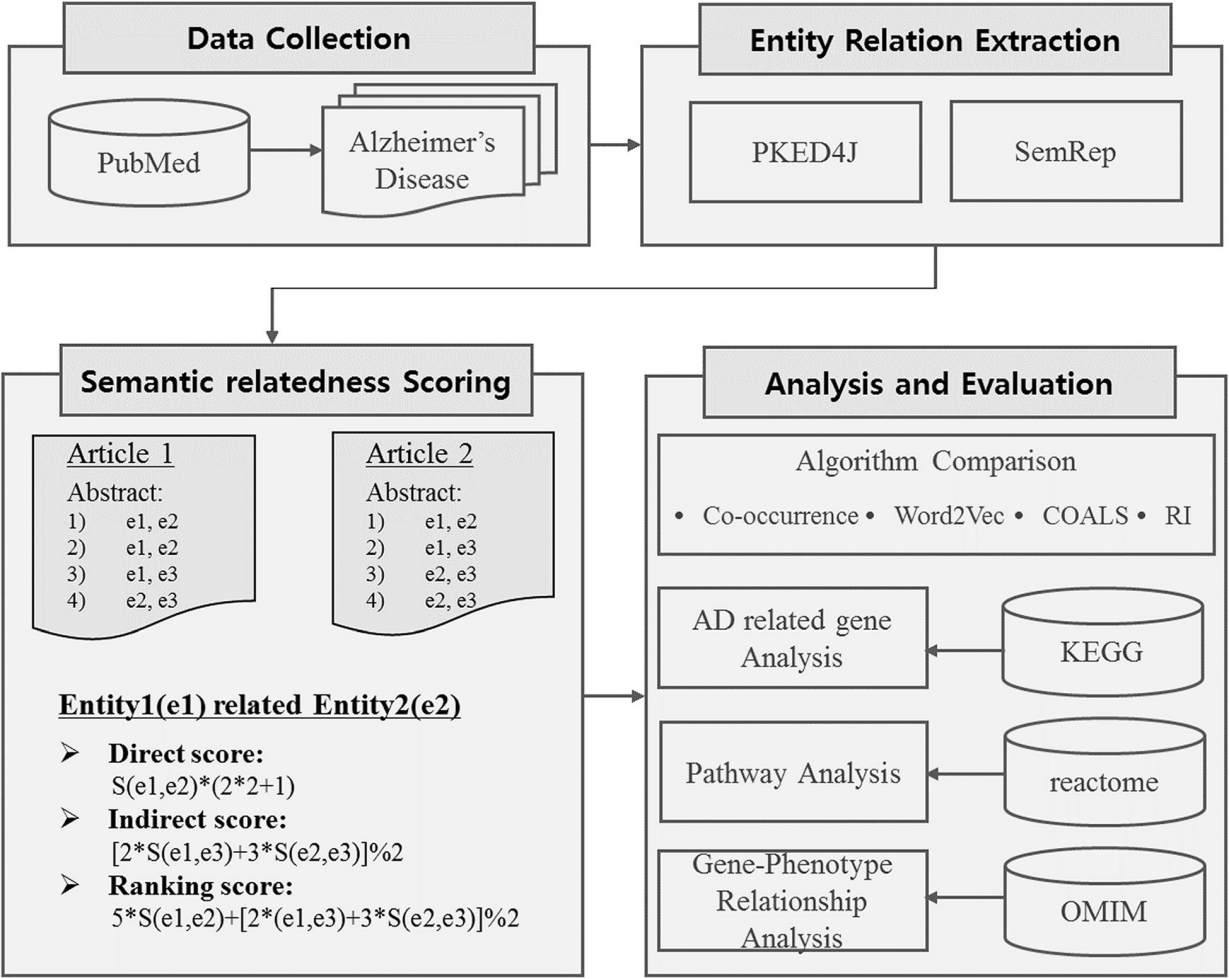
Overview of the proposed approach

The present study comprises four steps: data collection, entity relation extraction, semantic relatedness scoring calculation, and evaluation. For semantic relatedness scoring, we consider both direct and indirect connection; in terms of evaluation, we employ four kinds of analyses, namely algorithm comparison, AD related–gene analysis, pathway analysis, and gene–phenotype relation analysis. Figure 1 illustrates the overall design of this study. A detailed description of the proposed approach is provided in subsequent sections.

### Data collection

Using ‘Alzheimer disease’ or ‘Alzheimer’s disease’ as search terms, we retrieved 118,167 abstracts from PubMed, a search engine indexing more than 29 million citations for biomedical literature from MEDLINE. The exact query formulation is “Alzheimer disease [Title/Abstract] OR Alzheimer’s disease [Title/Abstract]”.

**Fig. 2:**
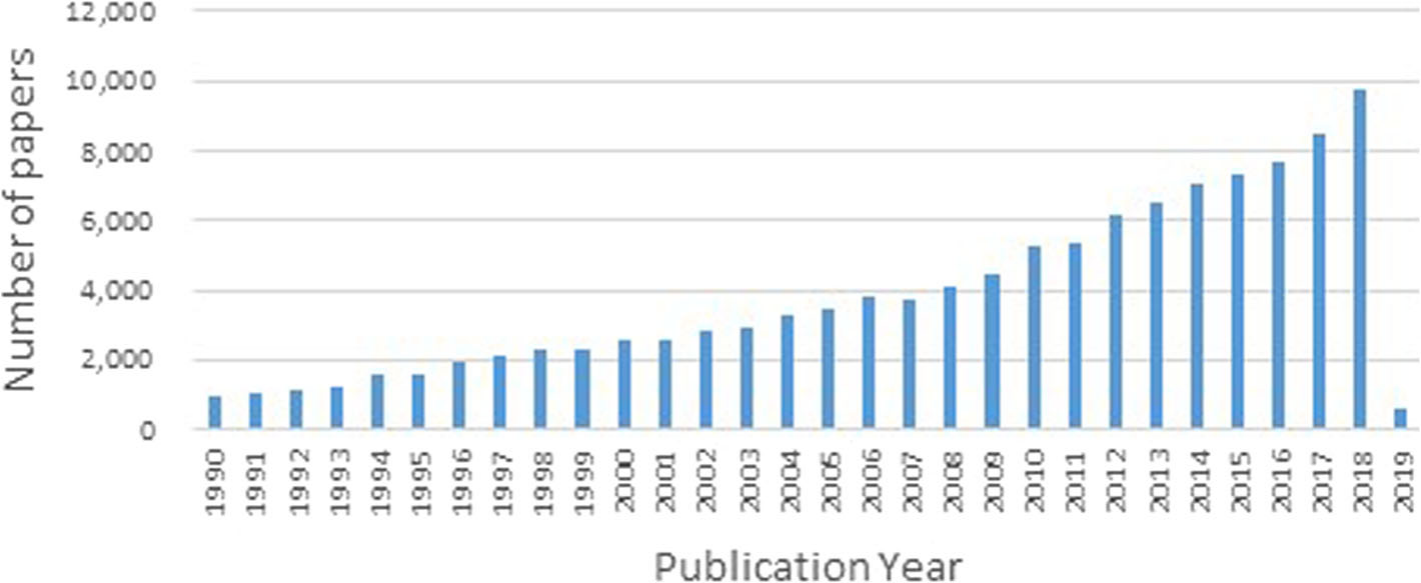
Number of papers by publication year

We did not limit publication by year, so as to get as much data as possible for our analysis. Figure 2 shows the distribution of the number of papers by publication year from 1990 to January 2019.

### Entity relation extraction

For PKDE4J [22], the algorithm used for entity relation extraction can identify the verb located between the two entities in a sentence and capture relational characteristics. In order to decrease unnecessary indirect connections, we selected entity by type. Since we focus on Alzheimer’s disease, we limited the entity type to gene, drug, and disease. Thus, for entity extraction, we used the following dictionaries: drug dictionaries, the gene dictionary collects from UniProt [29], MeSH (Medical Subject Headings) for disease [30], KEGG (Kyoto Encyclopedia of Genes and Genomes) for genetics [31], and DrugBank for medications [32]. We used the same data collection as the input for SemRep. As output, we extracted 969,341 entity relations using PKDE4J and 630,054 entity relations using SemRep [23].

### Semantic relatedness scoring calculation

We considered both direct and indirect scoring for each entity pair. For the direct score, after we extracted the relations of an entity pair, we looked at the same entity pairs with different relation types appearing in one abstract. An example is shown below: the first column is the PMID (PubMed unique identifier), the second column is sentence location in that abstract, and the last column is entity relations:

pmid | sentence location | entity1 | type | entity2 | type| relations.

19,395,124 | 8 | MCI | DISEASE | depression | DISEASE | CO-OCCUR |.

19,395,124 | 17 | MCI | DISEASE | depression | DISEASE | RESULT_OF |.

Next, we considered only the co-occurrence frequency of entity pairs. There are two different kinds of direct relations: 1) co-occurrence of an entity pair in one abstract with frequency greater than one as noted as ‘sum_same’ in Tables 1 and 2) one-time co-occurrence of an entity pair in one abstract as noted as ‘sum_different’ in Table 1. If an entity pair only co-occurs once in an abstract, the co-occurrence number is the same as the number of abstracts. Biomedical literatures, like any other literatures, have skewed distribution. In other words, much research tends to follow popular diseases, drugs, and genes. Due to this tendency, it is hard to identify a new relation by the co-occurrence method. Thus, we aim to find less visible information from biological texts. If two-entity pairs co-occur in several abstracts, it indicates these relations are more popular and we can infer they are well-known entity pairs. We give them a low weight, while assigning entity pairs found in the same abstract a higher weight. Table 1 represents pseudocode for our algorithm.

**Table 1:**
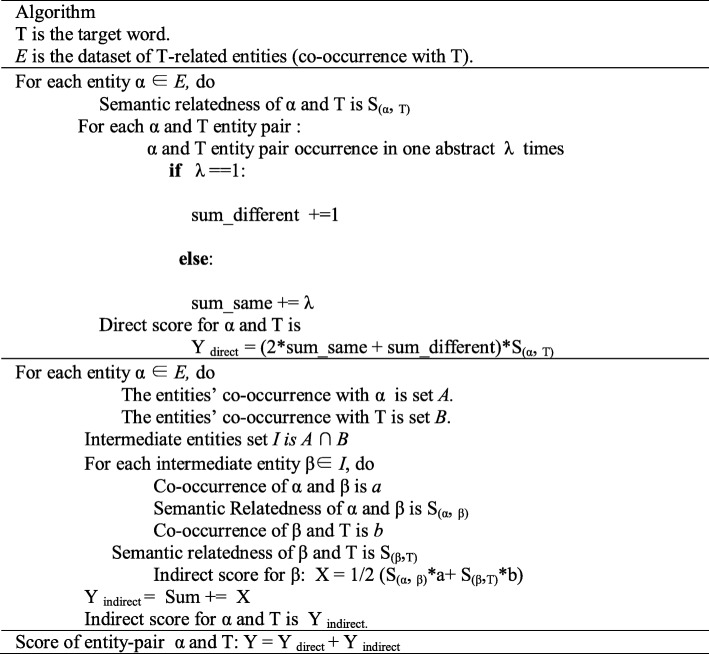
Pseudocode for our algorithm.

**Table 2:** Alzheimer’s disease–APP direct entity pairs

Entity(A)	Entity(C)	Direct Frequency	pmid_same	pmid_different	relatedness	Direct score (Y_direct_)
Alzheimer’s disease	APP	5126	4123	1003	0.427029	3949.592

Therefore, the direct score can be calculated as Formula (1):

1 $$ {\mathrm{Y}}_{\mathrm{direct}}={\mathrm{S}}_{\left(\mathrm{A},\mathrm{C}\right)}\ast \left(2\upalpha +\upbeta \right) $$

where S _(A, C)_ is a semantic relatedness score between entity A and entity C. The semantic relatedness score is the cosine similarity calculated by corpus- and thesaurus-trained word embedding, per Kiela et al. [18]. In their method, Kiela et al. use additional contexts (such as a thesaurus) to supplement the Skip-Gram. For each target word, they modify the object to include an additional context, so that each word is sampled uniformly from the set of additional contexts. In this case, the corpus consists of AD-related articles collected from PubMed, while our thesaurus is derived from PharmGKB’s Variant, Gene and Drug Relationship Data [33] and a gene synonym thesaurus from UniProt [29] used to construct a word embedding model for biological relations.

We denote by α the frequency of entity pairs that co-occur in one abstract more than once, while β is the frequency of entity pairs that co-occur in one abstract only once.

Table 2 shows the direct score of the (Alzheimer’s disease, APP) entity pair, where APP (for amyloid precursor protein) an Alzheimer’s-related gene.

Next, we consider the indirect score for each co-occurrence (Entity A, Entity C). This time we need to calculate the semantic relatedness score of each indirect entity pair using Formula (2), with the indirect semantic relatedness for each intermediate entity B defined as a weighted average of the direct semantic relatedness scores:

2 $$ {\mathrm{S}}_{\mathrm{indirect}\ \left(\mathrm{A},\mathrm{C}\right)}=\left[{\mathrm{S}}_{\left(\mathrm{A},\mathrm{B}\right)}\ast \mathrm{a}+{\mathrm{S}}_{\left(\mathrm{B},\mathrm{C}\right)}\ast \mathrm{b}\right]/\left(\mathrm{a}+\mathrm{b}\right) $$

where *a* and *b* are the co-occurrence frequencies between entities A and B and between entities B and C, respectively.

Then we calculate the indirect averaged semantic relatedness score over all possible intermediates B for the entity pair. We used indirect averaged similarity multiplied by the links count (*a* + *b*) for each indirect link path score X.

As shown in Table 3, the entity pair of Alzheimer’s disease and APP has 1834 “B” entities which are intermediate for them. Note that, for convenience, we only show five indirect link paths in Table 3. For example, if we apply the proposed method to (Alzheimer’s disease, BACE1), they co-occur 692 times (the *a* value), whereas (BACE1, APP) co-occurs 1294 times (the *b* value).

**Table 3:** Indirect entity pairs scores

Entity A	Co-occurrence (A, B)	relatedness (A, B)	Middle word B	Co-occurrence (B, C)	Relatedness (B, C)	Entity C	Score X
Alzheimer’s disease	1750	0.434575	PSEN1	1562	0.712967	APP	1874.1596
Alzheimer’s disease	692	0.398862	BACE1	1294	0.774334	APP	1278.0003
Alzheimer’s disease	3470	0.546675	amyloid beta	652	0.706621	APP	2357.6781
Alzheimer’s disease	471	0.449012	PSEN2	648	0.703564	APP	667.3944
Alzheimer’s disease	5107	0.464037	tau	526	0.628522	APP	2700.4406

Then we accumulate the score of all indirect link paths of two entities as the indirect score, using Formula (3):

3 $$ {Y}_{indirect}={\sum}_{i= 1}^n{X}_i={\sum}_{i= 1}^n\raisebox{1ex}{$ 1$}\!\left/ \!\raisebox{-1ex}{$ 2$}\right.\left[{S}_{\left(A,{B}_i\right)}\ast {a}_{\left(A,{B}_i\right)}+{S}_{\left({B}_i,C\right)}\ast {b}_{\left({B}_i,C\right)}\right] $$

where *n* is the number of indirect paths or the number of intermediate entities, *B*_*i*_ is the intermediate entity, ${S}_{\left(A,{B}_i\right)}$ is the semantic relatedness score between entities A and *B*_*i*,_ and ${S}_{\left({B}_i,C\right)}$ is the semantic relatedness score between entities *B*_*i*_ and C; ${a}_{\left(A,{B}_i\right)}$ is the co-occurrence frequency between A and *B*_*i*_, while ${b}_{\left({B}_i,C\right)}$ is the co-occurrence frequency between *B*_*i*_ and C.

Finally, we sum the direct score Y_direct_ and indirect score Y_indirect_ together as a semantic relatedness score for each entity pair (Formula (4)):

4 $$ \mathrm{Y}={\mathrm{Y}}_{\mathrm{direct}\ \left(\mathrm{formula}\ 1\right)}+{\mathrm{Y}}_{\mathrm{indirect}\ \left(\mathrm{formula}\ 3\right)} $$

where Y_direct_ and Y_indirect_ are calculated using Formulas (1) and (3), respectively.

## Results and discussion

To measure the performance of the proposed method, we compared it on the top 20 entity pairs with co-occurrence, Word2Vec similarity, COALS, and random indexing. Rohde et al. [24] proposed a model of semantic relatedness based on lexical co-occurrence, known as COALS. COALS is a vector space method for deriving word meanings from large corpora. First, co-occurrence counts are gathered. Next, common words are selected to create a co-occurrence matrix with word pair correlations converted to counts, setting negative values to 0 and taking square roots of positive values. After that, they summed the correlation of each word line in the matrix as the semantic similarity. Sahlgren [25] introduced a random indexing word space approach. Random indexing achieves high processing efficiency by only requiring a small amount of calculation. It uses context information to express the word vector of the characteristic word. However, the randomness of the vector elements (− 1, + 1, 0) may lead to additive subtraction in the calculation of feature word context vectors, with a resulting loss of potential semantic information. For comparison with the proposed method, we used COALS and random indexing to calculate semantic relatedness scores for each entity pair.

We analyzed the relation results between AD and genes by five methods: the proposed method, co-occurrence, Word2Vec [17], COALS [24], and random indexing [25]. In addition, we conducted pathway analysis and gene–phenotype relationship analysis to examine whether the proposed approach can be applied for other types of biological knowledge discovery.

### Top 20 entity pairs analysis

We calculated co-occurrence between entities extracted by PKDE4J. Table 4 shows the top 20 entities relating to Alzheimer’s disease by our proposed method. We used a min-max normalization method to generate each ranking score.

**Table 4:** Alzheimer’s disease top 20 related entity scores (PKDE4J)

No	Entity A	Entity C	Proposed	Co-occurrence	Word2Vec	COALS	Random indexing
1	Alzheimer’s disease	TAU	1	0.6181	0.607	0.6424	0.6188
2	Alzheimer’s disease	MCI	0.99	1	0.7571	0.1408	0.084
3	Alzheimer’s disease	Memory	0.9873	0.5843	0.6139	0.6618	0.6395
4	Alzheimer’s disease	Parkinson’s disease	0.935	0.5004	0.8704	1	1
5	Alzheimer’s disease	CSF	0.9072	0.4738	0.5717	0.1133	0.0547
6	Alzheimer’s disease	APP	0.9062	0.6204	0.5685	0.3317	0.2876
7	Alzheimer’s disease	APOE	0.8879	0.4328	0.606	0.1214	0.0633
8	Alzheimer’s disease	Neurodegenerative diseases	0.8689	0.4348	0.7678	0.11	0.0512
9	Alzheimer’s disease	Impairment	0.8035	0.1258	0.7224	0.9951	0.9948
10	Alzheimer’s disease	Amyloid beta	0.8024	0.4199	0.615	0.0777	0.0661
11	Alzheimer’s disease	Cognitive impairment	0.8002	0.1237	0.7617	0.1019	0.0426
12	Alzheimer’s disease	Neurodegeneration	0.7984	0.1464	0.7375	0.233	0.1823
13	Alzheimer’s disease	Neurodegenerative disorders	0.7863	0.2935	0.767	0.1521	0.0961
14	Alzheimer’s disease	Depression	0.7827	0.241	0.6844	0.4013	0.3617
15	Alzheimer’s disease	Oxidative stress	0.782	0.2512	0.6038	0.1084	0.0495
16	Alzheimer’s disease	Hippocampus	0.7794	0.0856	0.6091	0.1553	0.0995
17	Alzheimer’s disease	Vascular dementia	0.7683	0.3273	0.7796	0.6845	0.6636
18	Alzheimer’s disease	Patients	0.7589	0.016	0.9726	0.1235	0.0661
19	Alzheimer’s disease	Neurofibrillary tangles	0.7448	0.3975	0.6191	0.1553	0.0995
20	Alzheimer’s disease	MRI	0.7405	0.1315	0.5843	0.1472	0.0909

From Table 4, we can see that Tau (No. 1), CSF (No. 5), APOE (No. 7), and MRI (No. 20) have high semantic relatedness. In order to show the difference clearly we list the top 20 Alzheimer’s disease-related entities by each method in Table 5.

**Table 5:** Top 20 Alzheimer’s disease–related entities by each method (PKDE4J)

Entity A	Proposed
Alzheimer’s disease	[1] TAU [2] MCI [3] Memory [4] Parkinson’s disease [5] CSF [6] APP [7] APOE [8] Neurodegenerative diseases [9] Impairment [10] Amyloid beta [11] Cognitive impairment [12] Neurodegeneration [13] Neurodegenerative disorders [14] Depression [15] Oxidative stress [16] Hippocampus [17] Vascular dementia [18] Patients [19] Neurofibrillary tangles [20] MRI
Entity A	Co-occurrence
Alzheimer’s disease	[1] MCI [2] APP [3] TAU [4] Memory [5] Parkinson’s disease [6] CSF [7] Neurodegenerative diseases [8] APOE [9] Amyloid beta [10] Neurofibrillary tangles [11] Vascular dementia [12] Neurodegenerative disorders [13] Senile plaques [14] Oxidative stress [15] Neurodegenerative disorder [16] Depression [17] PD [18] PSEN1 [19] Amyloid plaques [20] Neurodegenerative disease
Entity A	Word2Vec
Alzheimer’s disease	[1] Asymptomatic Alzheimer’s disease [2] Alzheimer’s disease pathophysiology [3] Alzheimer’s disease neuropathology [4] Sporadic Alzheimer’s disease [5] Alzheimer’s disease patients [6] Early Alzheimer’s disease [7] Depression in Alzheimer’s disease [8] Late-onset Alzheimer’s disease [9] Incipient Alzheimer’s disease [10] Sporadic Alzheimer’s disease patients [11] Asymptomatic Alzheimer’s disease [12] Preclinical Alzheimer’s disease [13] Alzheimer’s disease dementia [14] Prodromal Alzheimer’s disease [15] Severe Alzheimer’s disease [16] Typical Alzheimer’s disease [17] Mild Alzheimer’s disease [18] Alzheimer’s disease with diabetes [19] Presenile Alzheimer’s disease [20] Familial Alzheimer’s disease
Entity A	COALS
Alzheimer’s disease	[1] Parkinson’s disease [2] Impairment [3] Vascular dementia [4] Memory [5]TAU [6] Neuronal [7] Increased [8] Dementias [9] Mild cognitive impairment [10] Huntington’s disease [11] Depression [12] Diabetes [13] Schizophrenia [14] Stroke [15] APP [16] Accumulation [17] Cancer [18] Down syndrome [19] Neurodegeneration [20] Caregivers
Entity A	Random indexing
Alzheimer’s disease	[1] Parkinson’s disease [2] Memory [3] TAU [4] Neuronal [5] Increased [6] Dementias [7] Mild cognitive impairment [8] Impairment [9] Vascular dementia [10] Biomarkers [11] Huntington’s disease [12] Depression [13] Diabetes [14] Schizophrenia [15] Stroke [16] APP [17] Accumulation [18] Cancer [19] Downs syndrome [20] Neurodegeneration

As shown in Table 5, we can see that for Tau (No. 1), CSF (No. 5), APOE (No. 7), cognitive impairment (No. 11), and MRI (No. 20) the proposed method achieves a higher ranking than other methods. Specifically, among the top 20 entities lists, MRI (magnetic resonance imaging) only appears in our proposed method. This is attributed to the fact that these entities are either core proteins, genes related to AD, or diagnostic methods for AD, all of which may have many intermediate entities helping them link with AD so that they tend to gain a higher semantic relatedness score. Tau protein is a microtubule-associated protein (MAP) involved in microtubule stabilization. It is also a multifunctional protein that plays a key role in certain neurodegenerative diseases such as AD [34]. AD and Tau have 3568 co-occurrences in our dataset, with 236 different intermediate entities to help them link together. For CSF (cerebrospinal fluid), there is strong evidence that special CSF tests may be helpful in diagnosis. AD and CSF have 1968 co-occurrences, with 288 different intermediate entities. APOE gene polymorphism is closely related to AD, coronary heart disease, hyperlipidemia, cerebral infarction, and other diseases. Through the detection of APOE gene type, the incidence probability of senile dementia, cardiovascular and cerebrovascular diseases, and other diseases can be predicted at an early stage, to achieve early detection and intervention and to maximize a patient’s survival period. Studies have found that APOE is closely related to the incidence of AD, and the E4 allele of APOE is a high-risk factor for AD, especially in female patients [24]. AD and APOE entity pairs have 1401 intermediate entities to link them together.

While some entities rank higher by other methods, senile plaques only show in the co-occurrence top 20 results. The top Word2Vec results are all phrases containing “Alzheimer’s disease.” Regarding COALS and random indexing methods, the COALS-ranked terms Huntington’s disease (No. 10), diabetes (No. 12), schizophrenia (No. 13), and stroke (No. 14) only appear in these two rankings. COALS and RI seem to have better performance, yet their calculation principles allow the top 20 entities to have almost identical semantic relatedness scores; thus, it is hard to use COALS and RI to rank the entities.

We also examined the top 50 entities by each method, omitted here due to space limitations; the results are publicly available at http://informatics.yonsei.ac.kr/semantics/Top_50_entity_pair_result.xlsx.

For the top 20 entities, the APOE gene is 7th by our method. However, the APOE gene is not shown by COALS, Word2Vec, or random indexing in the top 20 ranking list. For the top 50 entities, dementia with Lewy bodies and FTD (frontotemporal dementia) are ranked high only by our proposed method. Alzheimer's disease, vascular dementia, and Lewy body dementia are seen as the top three most common causes of dementia. However, memantine (a drug) is shown by co-occurrence only. Multiple sclerosis (MS; a disease) only appears through COALS and random indexing.

Regarding the SemRep results, since we did not select the entity type, there are many words in common in the top 20 and top 50 lists. For example, as shown in Table 6, brain (No. 3), Alzheimer’s disease can affect memory in the patient’s brain; these entity pairs are already well known.

**Table 6:** Alzheimer disease top 20 related entity scores (SemRep)

No	Entity A	Entity C	Ranking score	Co-occurrence	Word2Vec	COALS	Random indexing
1	Alzheimer’s disease	Patients	1	1	0.5444	0.0413	0.0206
2	Alzheimer’s disease	Disease	0.9065	0.1167	0.98	0.8615	0.8585
3	Alzheimer’s disease	Brain	0.5989	0.023	0.6085	0.1052	0.0859
4	Alzheimer’s disease	Dementia	0.5902	0.1187	0.733	0.6591	0.6518
5	Alzheimer’s disease	Impaired cognition	0.5013	0.0637	0.6737	0.0413	0.0206
6	Alzheimer’s disease	Therapeutic procedure	0.4828	0.0512	0.6606	0.0413	0.0206
7	Alzheimer’s disease	Neurodegenerative disorders	0.4371	0.0444	0.7707	0.0413	0.0206
8	Alzheimer’s disease	Persons	0.408	0.0879	0.6314	0.0413	0.0206
9	Alzheimer’s disease	Amyloid	0.4018	0.0357	0.6118	0.1159	0.0968
10	Alzheimer’s disease	Pharmaceutical preparations	0.394	0.03	0.7011	0.0413	0.0206
11	Alzheimer’s disease	APP gene	0.3845	0.0098	0.5346	0.0413	0.0206
12	Alzheimer’s disease	Amyloid beta-protein precursor	0.3751	0.0211	0.3492	0.0413	0.0206
13	Alzheimer’s disease	Functional disorder	0.3746	0.0354	0.7702	0.0413	0.0206
14	Alzheimer’s disease	Apolipoprotein E	0.3707	0.0292	0.5656	0.052	0.0315
15	Alzheimer’s disease	Parkinson’s disease	0.3702	0.0052	0.9802	0.0413	0.0206
16	Alzheimer’s disease	Population group	0.3692	0.0464	0.629	0.0413	0.0206
17	Alzheimer’s disease	Pathogenesis	0.3685	0.0405	0.7519	0.0413	0.0206
18	Alzheimer’s disease	Dementia, vascular	0.3552	0.0095	0.7878	0.6494	0.5067
19	Alzheimer’s disease	Nerve Degeneration	0.3546	0.0282	0.712	0.0413	0.0206
20	Alzheimer’s disease	Entire hippocampus	0.3541	0.0038	0.5782	0.0413	0.0206

As shown in Table 7, APP gene (No. 11), Apolipoprotein E (No. 14), and Parkinson’s disease (No. 15) have a higher score by the proposed method than by the other methods, due to intermediate entities. The top 20 entities in the Word2Vec ranking are all disease-related entities. However, there are many drug names that only appear in random indexing methods, such as donepezil (No. 6) and rivastigmine (No. 17).

**Table 7:** Top 20 Alzheimer’s disease–related entities by each method (SemRep)

Entity A	Proposed
Alzheimer’s disease	[1] Patients [2] Disease [3] Brain [4] Dementia [5] Impaired cognition [6] Therapeutic procedure [7] Neurodegenerative disorders [8] Persons [9] Amyloid [10] Pharmaceutical preparations [11] APP gene [12] Amyloid beta-protein precursor [13] Functional disorder [14] Apolipoprotein E [15] Parkinson’s disease [16] Population group [17] Pathogenesis [18] Dementia, vascular [19] Nerve degeneration [20] Entire hippocampus
Entity A	Co-occurrence
Alzheimer’s disease	[1] Patients [2] Dementia [3] Disease [4] Persons [5] Individual [6] Impaired cognition [7] Therapeutic procedure [8] Population group [9] Neurodegenerative disorders [10] Elderly [11] Pathogenesis [12] Amyloid [13] Senile plaques [14] Functional disorder [15] Pharmaceutical preparations [16] Apolipoprotein E [17] Nerve degeneration [18] Brain [19] Participant [20] Woman
Entity A	Word2Vec
Alzheimer’s disease	[1] Tangier disease [2] Lyme disease [3] Binswanger disease [4] Parkinson’s disease [5] Disease [6] Huntington’s disease [7] Autosomal recessive juvenile Parkinson’s disease [8] Disease progression [9] Alzheimer’s disease, late onset [10] Alzheimer’s disease, early onset [11] Familial Alzheimer’s disease [12] Progressive disease [13] Alzheimer’s disease assessment scale [14] Genetic predisposition to disease [15] Chronic disease [16] Pick disease of the brain [17] Disease model [18] Chronic obstructive airway disease [19] Psychic disease [20] Motor neurone disease
Entity A	COALS
Alzheimer’s disease	[1] Response [2] Patients [3] Impaired cognition [4] Therapeutic procedure [5] Neurodegenerative disorders [6] Persons [7] Pharmaceutical preparations [8] APP gene [9] Amyloid beta-protein precursor [10] Functional disorder [11] Parkinson’s disease [12] Population group [13] Pathogenesis [14] Nerve degeneration [15] Entire hippocampus [16] Memory impairment [17] Senile plaques [18] Proteins [19] Nervous system disorder [20] Genes
Entity A	Random indexing
Alzheimer’s disease	[1] Response [2] Inhibitors [3] Cohort [4] Tomography [5] Disease [6] Donepezil [7] Neuroimaging [8] Receptor [9] Dementia [10] Dementia, vascular [11] Presenilin [12] Network [13] Presenilin-1 [14] Follow-up [15] Sex [16] DNA [17] Rivastigmine [18] Investigation [19] Density [20] Hyperphosphorylation

### Alzheimer’s disease-related gene analysis

**Fig. 3:**
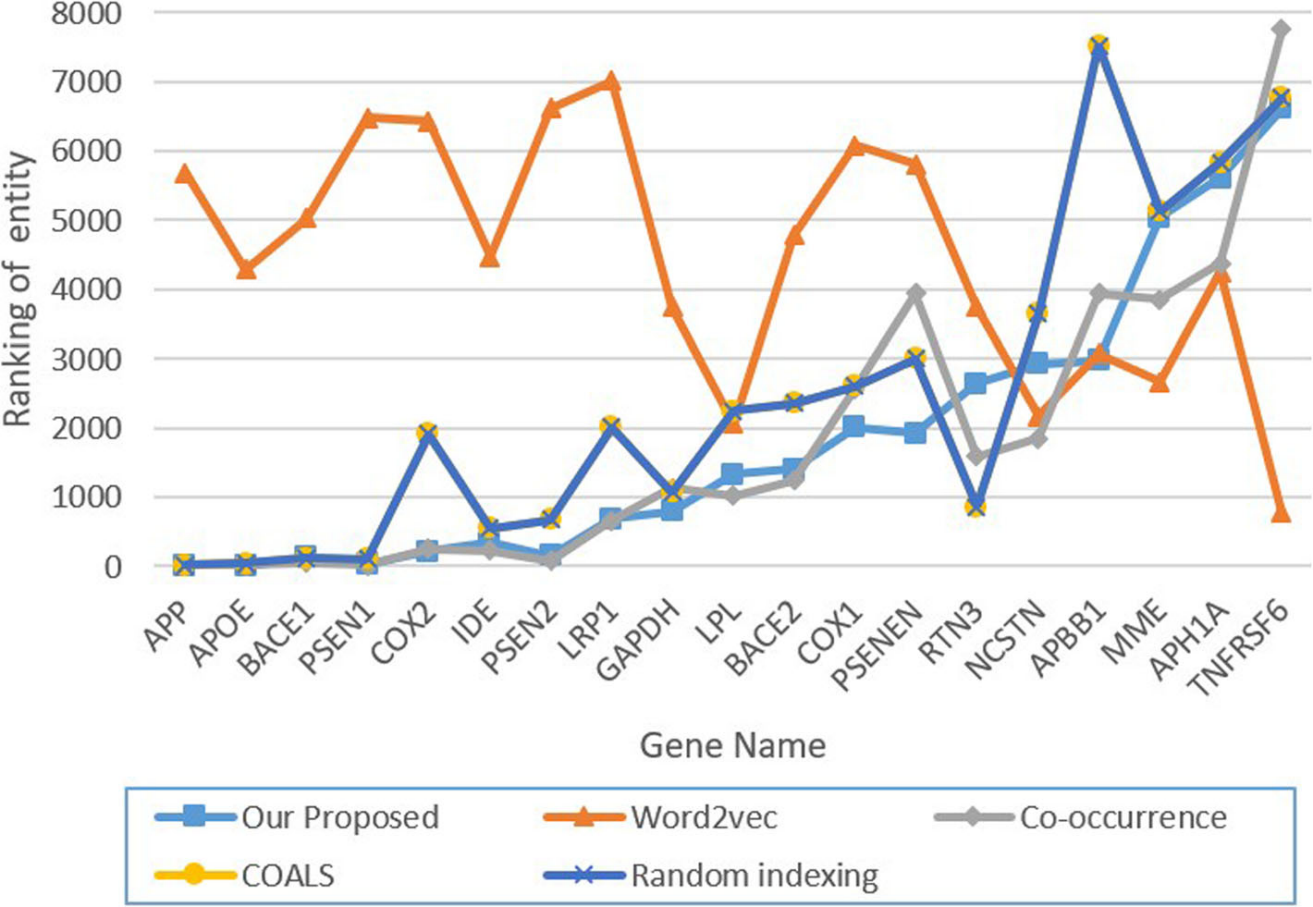
Alzheimer’s disease–related gene ranking (PKDE4J)

For the PKDE4J results, we identified 8696 entities which co-occur with Alzheimer’s disease. For evaluation, we collected the related genes for Alzheimer’s from KEGG and calculated the ranking of each rated gene using co-occurrence frequency, our ranking method, and Word2Vec [17], COALS [24], and random indexing [25]. Figure 3 shows the Alzheimer’s disease-related gene ranking in each ranking list. The horizontal axis shows the gene name, while the vertical axis shows the ranking of each entity.

Since the vertical axis shows the ranking for each gene, a small number means the ranking is high. With Word2Vec, gene rankings are always lower than for other methods; the COALS and random indexing methods have similar gene rankings. For COX1 and PSENEN genes, our method shows a higher ranking than the others. For the (AD, COX1) entity pair, the number of co-occurrences is only 6 in our database. However, there are 72 different intermediate entities to help them link together. For the (AD, PSENEN) entity pair, the number of co-occurrences is only 3 in our dataset with 12 intermediate entities (so there are 12 indirect paths).

We summarize the results of the gene analysis shown in Fig. 3 in Table 8. At first sight, our proposed method seems not much different from co-occurrence, COALS, and random indexing, but these methods share the weakness that many entities have the same score. Thus, it is hard to interpret these entities’ rankings effectively.

**Table 8:** Comparison of Alzheimer’s disease–related gene ranking (PKDE4J)

Pair rank	Co-occurrence	COALS	Random indexing	Word2Vec	Proposed
1–10	1	0	0	0	1
11–100	2	2	2	0	1
101–500	4	1	1	0	5
501–2000	6	6	6	1	5
2000–3999	2	5	5	6	4
4000–5999	3	2	2	7	2
6000–9000	1	1	1	5	1

In the PKDE4J result, with 19 AD-related genes, we found 8696 entities co-occurring with AD. To analyze how many AD-related genes occur in the top 20% of (top 1740 ranked) entities, we calculated precision, recall, and F-measure for each method. As shown in Table 9, our proposed method achieved the joint highest F-measure of 65.94% together with the co-occurrence method.

**Table 9:** Quantitative evaluation in PKDE4J

Method name	Precision*10^**2**^	Recall	F-Measure
Proposed	68,97%	63.16%	65.94%
Word2Vec	5.74%	5.26%	5.30%
Co-occurrence	68,97%	63.16%	65.94%
COALS	51.72%	47.37%	49.45%
RI	45.98%	42.10%	43.95%

### Pathway analysis

In bioinformatics research involving an intricate network of interactions, pathways analysis is often quite useful. Pathways can help to explain gene function in the context of biological processes.

**Fig. 4:**
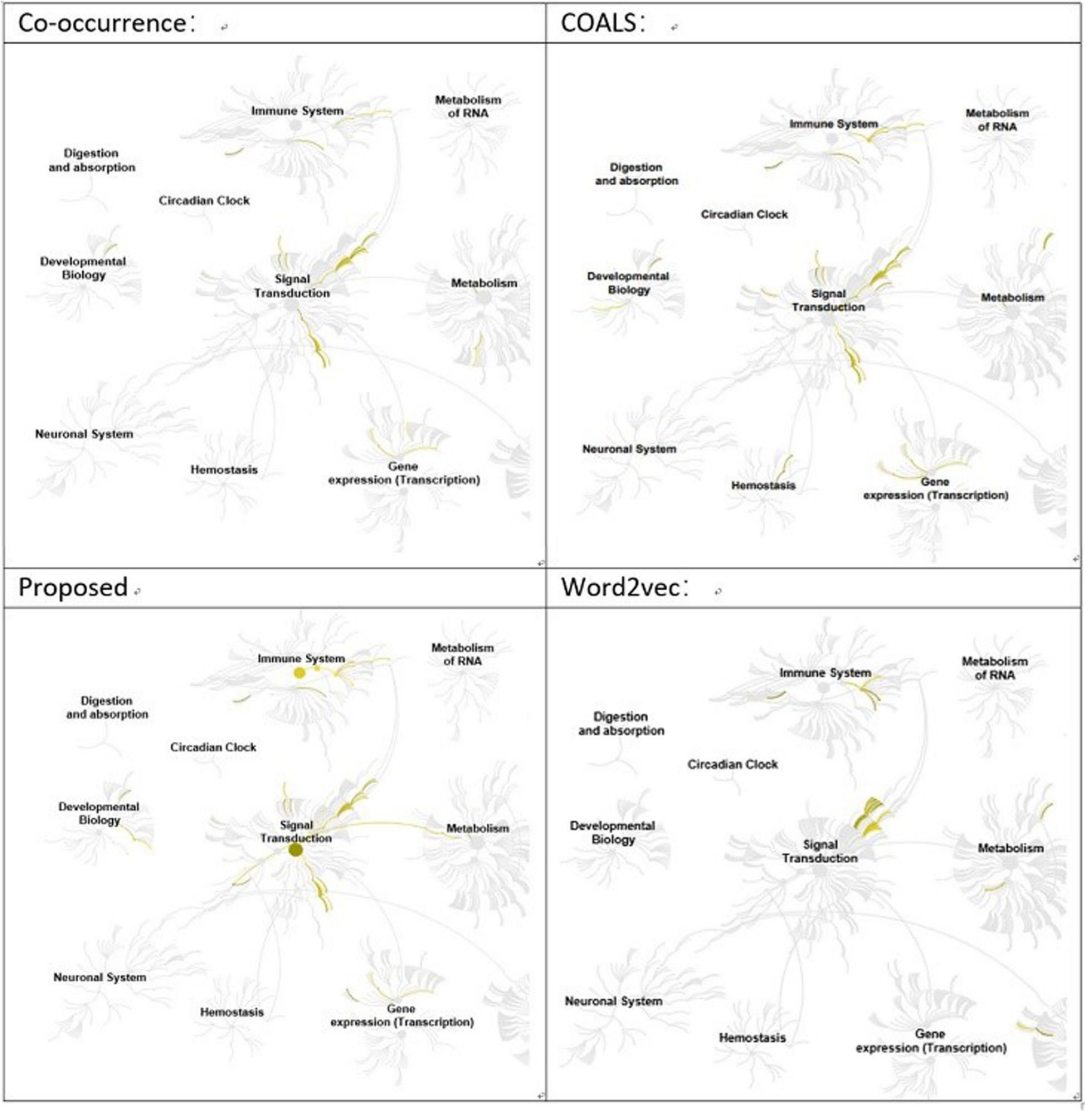
Genome-wide overview

We applied the proposed method, co-occurrence, COALS [24], and Word2Vec [17] to select the top 20 genes in each ranking list, and used Reactome to do pathway analysis; Fig. 4 shows a genome-wide overview for each method.

Figure 4 shows that a series of genes are involved in pathways. The yellow marks are pathways that at least have one of the genes in our gene list. Pathways in Reactome are arranged in a hierarchy, the center of each cluster being the root of one top-level pathway. Each step away from the center represents the next level lower in the pathway hierarchy.

In this genome-wide overview, Word2Vec has fewer pathways than the other methods. In the developmental biology cluster, only the proposed method and COALS have related pathways. However, in the hemostasis cluster, only COALS has genes which can be mapped to pathways. In addition, in the signal transduction and immune system clusters, our proposed method has pathways from the root or top-level pathway, and also has a pathway which connects the signal transduction cluster to the metabolism cluster.

The top 20 gene list genome-wide overview shows that from the pathway perspective, our proposed method has better performance for genes identified in (functional) cross clustering and higher-level pathways.

We now analyze these pathways in detail. Table 10 shows the top five most significant pathways by *p*-value in each gene list [26]. “Entities” are the input genes. “Reactions” can be regarded as the ‘steps’ of pathways: any biological event that changes the state of a biological molecule. “Entities found” is the number of common entities between the submitted data set and the pathway. “Entities ratio” is the proportion of pathway molecules represented by this pathway. “p-value” is the result of statistical testing for over-representation of entities. “Reactions found” counts pathways with at least one molecule in the submitted data set represented. “Reactions ratio” is the proportion of all reactions represented by reactions from this pathway.

**Table 10:** Top five important pathways sorted by p-value for each gene list

Proposed						
Pathway name	Entities				Reactions	
	found	ratio	p-value	FDR*	found	ratio
Nuclear signaling by ERBB4	3 / 35	0.002	5.37e-05	0.012	3 / 22	0.002
Signaling by interleukins	7 / 640	0.046	2.60e-04	0.019	6 / 491	0.041
MECP2 regulates transcription of neuronal ligands	3 / 61	0.004	2.74e-04	0.019	3 / 37	0.003
Signaling by receptor tyrosine kinases	2 / 13	9.25e-04	3.41e-04	0.019	2 / 8	6.68e-04
NRIF signals cell death from the nucleus	6 / 521	0.037	5.89e-04	0.023	71 / 633	0.053
Co-occurrence						
MECP2 regulates transcription of neuronal ligands	2 / 13	9.25e-04	3.18e-04	0.036	2 / 8	6.68e-04
RUNX1 and FOXP3 control the development of regulatory T lymphocytes (Tregs)	2 / 17	0.001	5.41e-04	0.036	2 / 20	0.002
NRIF signals cell death from the nucleus	2 / 18	0.001	6.06e-04	0.036	4 / 7	5.84e-04
Amyloid fiber formation	3 / 88	0.006	7.15e-04	0.036	16 / 33	0.003
Neurodegenerative diseases	2 / 30	0.002	0.002	0.056	2 / 22	0.002
COALS						
Plasma lipoprotein assembly	3 / 30	0.002	4.18e-05	0.01	8 / 19	0.002
MECP2 regulates transcription of neuronal ligands	2 / 13	9.25e-04	3.91e-04	0.042	2 / 8	6.68e-04
HDL assembly	2 / 18	0.001	7.44e-04	0.042	7 / 9	7.51e-04
NRIF signals cell death from the nucleus	2 / 18	0.001	7.44e-04	0.042	4 / 7	5.84e-04
Amyloid fiber formation	3 / 88	0.006	9.67e-04	0.044	16 / 33	0.003
Word2Vec						
Transfer of LPS from LBP carrier to CD14	1 / 3	2.13e-04	0.005	0.075	2 / 2	1.67e-04
NTF3 activates NTRK2 (TRKB) signaling	1 / 4	2.85e-04	0.006	0.075	3 / 3	2.50e-04
NTF4 activates NTRK2 (TRKB) signaling	1 / 4	2.85e-04	0.006	0.075	3 / 3	2.50e-04
BDNF activates NTRK2 (TRKB) signaling	1 / 4	2.85e-04	0.006	0.075	3 / 3	2.50e-04
Defective GSS causes glutathione synthetase deficiency (GSS deficiency)	1 / 4	2.85e-04	0.006	0.075	1 / 1	8.34e-05

* False Discovery Rate

We can see that our proposed method has a higher probability that many entities are found in the same pathways. For example, “Signaling by interleukins” has 7 genes (input gene is 20). However, the gene list selected by Word2Vec has greater dispersion. This may imply that if a disease-related gene is over-represented in the same pathway, then other genes in that pathway may have an impact on the disease.

Table 11 shows the gene rankings by five methods for the top 10 gene rankings by number of pathways.

**Table 11:** Gene rankings for each method, ordered by pathway number

Extraction		Ranking Numbers					
	Gene	Proposed	Co-occurrence	Word2Vec	COALS	Random Indexing	Pathway
PKDE4J	*TNF	361	742	4876	548	851	57
	*EGFR	1468	2063	5550	2391	3381	43
	*GSK3B	990	1374	2341	2119	2349	39
	*CREB1	6298	8240	6792	6468	7460	39
	IL1B	2401	1292	3451	3043	3733	37
	*SRC	3854	8230	3984	4257	5257	36
	*ATF4	2890	6965	4204	3430	4538	32
	MYC	6531	4909	4957	6688	6578	32
	*IGF1	446	463	3410	1940	2040	30
	IGF1R	1868	1397	3697	519	1119	29
SemRep	MAP 2 K1	3466	3462	3433	1.5	1.2	80
	PRKACB	3952	2131	3444	1.5	1.2	67
	*MAPK8	253	1004	3410	1.5	1.2	59
	*TNF	731	3564	2460	1.5	1.2	57
	JUN	2428	1804	2799	1.5	1.2	49
	*TP53	399	724	2492	1.5	1.2	48
	*IL6	925	1012	2816	1.5	1.2	43
	EGFR	3201	3032	2497	1.5	1.2	43
	*BAX	2099	3643	2780	1.5	1.2	41
	IL1B	2264	1910	2246	1.5	1.2	37

The genes with the asterisk (*) symbol indicate that our method generates better ranking than the other methods do

We summarize the results as shown in Table 11 as Table 12. Our proposed method has clear advantages in selecting genes that can act through more pathways. Genes in the same pathway may have proximate gene expression. Gene expression provides a fundamental basis for genotype to trigger phenotype. Our analysis seems to imply that similar gene expressions may have a homogeneous impact on gene–phenotype association. These genes with similar phenotype associations tend to have a higher chance of co-occurrence in the biological literature. Since our method also considers indirect relations, which can help to link these co-occurrences, the genes which participate in many pathways get higher scores by our method.

**Table 12:** Top 10 genes ordered by pathway number

System	Co-occurrence	COALS	Random indexing	Word2Vec	Proposed
PKDE4J	3	0	0	0	7
SemRep	3	–	–	2	5

### Gene–phenotype relationship analysis

Phenotype is the result of comprehensive regulation of molecular events at all levels. Different genotypes can produce the same phenotype, while the same genotype can produce different phenotypes, which makes the scientific problem of genetic regulation from genotype to phenotype highly complex. Therefore, studying the genotype-to-phenotype aspect of genetic regulation is of critical scientific significance, particularly as the biological literature continues to grow exponentially. Genes with the same phenotype are more likely to be researched in one paper, which increases the possibilities for co-occurrence. Our proposed method considers both direct and indirect relations and semantic relatedness for entity pairs, which makes it easier to find genes controlling the same phenotype; this kind of knowledge discovery can help biologists to find new regulatory pathways and mechanisms. Moreover, summarizing the genetic “rules” of disease allows targeting to improve prevention, treatment, and comprehensive measures to reduce morbidity.

For example, the presence of the APOE4 allele is strongly associated with the onset of early-onset familial Alzheimer’s disease. The APOE4 allele is also an important gene for coronary artery disease; in other words, APOE4 has an impact on two phenotypes. Therefore, Alzheimer’s disease and coronary artery disease may share some relations.

Table 13 shows the indirect score for Alzheimer’s disease and coronary artery disease entity pairs by our proposed method. We show the top 20 results by co-occurrence between intermediate entity B and entity C.

**Table 13:** Indirect relations for Alzheimer’s disease and coronary artery disease

Entity A	Co-occurrences of (A, B)	Relatedness	Intermediate entity B	Co-occurrences of (B, C)	Relatedness	Entity C
Alzheimers disease	1128	0.65167	Diabetes	14	0.75549	Coronary artery disease
Alzheimers disease	409	0.61262	Hypertension	13	0.78521	Coronary artery disease
Alzheimers disease	687	0.46660	Cholesterol	10	0.54389	Coronary artery disease
Alzheimers disease	1789	0.49688	APOE	9	0.55493	Coronary artery disease
Alzheimers disease	452	0.68793	Atherosclerosis	7	0.81259	Coronary artery disease
Alzheimers disease	437	0.65669	Type 2 diabetes	7	0.74681	Coronary artery disease
Alzheimers disease	1166	0.73817	Schizophrenia	6	0.66643	Coronary artery disease
Alzheimers disease	408	0.66002	Diabetes mellitus	6	0.75417	Coronary artery disease
Alzheimers disease	28	0.64372	Atrial fibrillation	6	0.70951	Coronary artery disease
Alzheimers disease	147	0.69426	Bipolar disorder	4	0.66286	Coronary artery disease
Alzheimers disease	49	0.66373	Heart failure	4	0.78867	Coronary artery disease
Alzheimers disease	27	0.52386	PON1	4	0.59528	Coronary artery disease
Alzheimers disease	4135	0.97112	Parkinson’s disease	3	0.84996	Coronary artery disease
Alzheimers disease	1992	0.58868	Depression	3	0.55049	Coronary artery disease
Alzheimers disease	357	0.63473	Obesity	3	0.72262	Coronary artery disease
Alzheimers disease	221	0.50534	APOE4	3	0.54360	Coronary artery disease
Alzheimers disease	165	0.73497	Osteoporosis	3	0.75669	Coronary artery disease
Alzheimers disease	58	0.64033	Genome-wide association study	3	0.65748	Coronary artery disease
Alzheimers disease	4414	0.66884	Mild cognitive impairment	2	0.61373	Coronary artery disease

APOE has high co-occurrence with these two diseases, implying that our method can be used to find the related genes for a given phenotype.

We collect the phenotypes of Alzheimer’s disease co-occurrence genes from the OMIM database, ranking by number of common-phenotype genes. Table 14 shows an example. The second column is the co-occurrence gene. The last column is the number of genes.

**Table 14:** Gene–phenotype ranking by number of common-phenotype genes (PKDE4J)

Entity A	Entity C (Gene Only)	Phenotype	Phenotype-Related Gene	Common Phenotype Genes
Alzheimers disease	CD36	Platelet glycoprotein IV deficiency	CD36	17
		Macrothrombocytopenia	–	
		Coronary heart disease	CD36	
		Malaria, cerebral	ACKR1, FCGR2A, FCGR2B, FCGR2A, FCGR2B, CR1, GYPC, CISH, GYPB, GYPA, TNF, HBB, TIRAP, NOS2A, SLC4A1, ICAM1,G6PD, CD36	
Alzheimers disease	IL10	Graft-versus-host disease	IL10	13
		HIV-1	CXCR1, CX3CR1, TLR3, HLAC, CXCL12, IFNG, IL4R, CCL3L1, CCL2, CCL11, CCL3, CD209, KIR3DL1, IL10	
		Rheumatoid arthritis	–	
Alzheimers disease	ABCA1	HDL deficiency	APOA1, ABCA1	2
		Tangier disease	ABCA1	
		Coronary artery disease, familial	LDLR, ABCA1	

In Table 15, we compare the top 10 gene rankings, ranked by number of common phenotype genes, and can clearly see that our proposed method has obvious advantages.

**Table 15:** Top ten gene rankings, ranked by number of common-phenotype genes

System	Co-occurrence	COALS	Random indexing	Word2Vec	Proposed
PKDE4J	2	0	0	2	6
SemRep	0	–	–	2	8

## Conclusion

With the growth in biomedical literature, how to identify meaningful information effectively from this literature becomes a crucial question. In this paper, we proposed a new semantic relatedness scoring algorithm for entity pairs by incorporating co-occurrence with consideration of both direct and indirect relations via specialized word embeddings. In addition, we used corpus and thesaurus to train word embeddings in order to calculate the semantic relatedness of each entity pair for ranking. We conducted evaluation in four ways:

1. We analyzed the top 20 and 50 entities ranked by our proposed method and compared them with co-occurrence, Word2Vec, COALS, and random indexing. The proposed method was able to select the entities that not only highly co-occur but also have more indirect relations for the target entity (in this paper, we used Alzheimer’s disease). For example, the APOE gene is top-ranked by our method but not by the other methods.
2. We collected the Alzheimer’s disease related genes from the KEGG database and examined them for ranking positions generated by the five approaches. Our method does not have a great advantage over the others, but it does generate distinct scores for entity pairs, whereas the other methods such as COALS and random indexing produce the same ranking scores, making it difficult to differentiate the degree of association of one entity pair from another.
3. We adopted pathway analysis for the top 20 genes listed by four different methods. Pathways allow us a macro perspective on the gene list. Our proposed method achieves better performance at identifying (functional) cross clustering as well as higher-level pathways.
4. We also conducted gene–phenotype relationship analysis to examine whether our method has an advantage. We found that the APOE4 gene plays a role in two phenotypes: Alzheimer’s disease and coronary artery disease. The results show that an indirect relation exists between the common gene and these two phenotypes. This means that if phenotypes are given, their common genotype can be identified by our method, which helps to uncover the genetic laws of heredity disease, and can offer better treatments.

This study has two major limitations. First, selection of entity type in the SemRep results was not properly done so as to reduce unnecessary indirect relations. Due to this, the most common entity, brain, achieves a higher score by our ranking method, which considers indirect relations, than by the other methods. Another major limitation is the lack of in-depth analysis of pathway. In a follow-up study, we plan to conduct laboratory experiments on the results identified by the proposed method. In addition, we plan to improve the quality of semantic relatedness scores by incorporating other lexical properties and contextual information for entities buried in biomedical literatures.

## Data Availability

Not applicable.

## Abbreviations

AD: Alzheimer’s disease
APP: Amyloid precursor protein
COALS: Correlated occurrence analog to lexical semantics
CSF: Cerebrospinal fluid
FTD: Frontotemporal dementia
IE: Information extraction
KEGG: Kyoto encyclopedia of genes and genomes
LCS: Least Common Subsumer
LDA: Latent dirichlet allocation
LSI: Latent semantic indexing
MAP: Microtubule-associated protein
MeSH: Medical subject headings
MRI: Magnetic resonance imaging
NLM: National library of medicine
NLP: Natural language processing
OMIM: Online mendelian inheritance in man
PMID: PubMed unique identifier
RI: Random indexing
UMLS: Unified medical language system
